# Alcohol as a Medicine

**Published:** 1867-10

**Authors:** W. M. Cornell

**Affiliations:** Boston, Mass.


### Correspondence.

### Alcohol as a Medicine.

BY W. M. CORNELL, M. D., LL.D., BOSTON, MASS.

Dr. Miner:—I have not forgotten my promise in your September number, to send you some "cases" of atomised inhalation, etc., but, if you will accept this article as a substitute for the present, the others, (D.V.) shall come. My attention is called to this subject now by the article "On the Indiscriminate Use of Alcoholic Stimulants in Disease," by Dr. Samuel Wilks, in that issue. I agree with Dr. Wilks, that "the most important question in therapeutics at the present day is the value of alcohol in disease;" and, I feel the more inclined to say a word on this matter because, in a little book on "Consumption Treated," published seventeen years ago, I spoke particularly *on the use of alcohol in phthisis.* At that time, it may be remembered, nothing, or next to nothing, had been published by the profession, on that subject. As the book is now out of print, save a single copy, you will pardon me in making the following quotation:

"I have seen consumptive patients greatly benefited by alcohol. I hope none of the friends of temperance will feel alarmed at this remark, as I speak of it *wholly as a medicine in this disease.* Long before I was able in any way to account for it, I found it to be a fact, that *spirit-drinkers* were much more apt to recover from *lung* attacks than any other class in the community. The fact, I knew; the why, I could not tell. But, the recent investigations upon pathology and therapeutics appear to me to have thrown some light upon this subject.

"Many morbid anatomists, among whom Dr. Bennett holds a prominent place, have asserted that *puckerings* or *cicatrices* of the lungs—indicating tubercular disease at some former period, which had been cured—are much oftener found in the dead bodies of spirit-drinkers, who have died of other diseases, than of others. This being the fact, may not the following be the reason: alcohol prevents the *arterialization* of the blood, as it circulates in it *unchanged*, uniting with the oxygen in the blood, and forming with it carbonic acid, thus keeping the blood in a *venous* condition, and preventing that abundance of *fibrin* upon which consumption depends. It has long been known that alcohol is not *digested;* and chemists tell us that it has a constant tendency to keep the blood *venous*.

"Now, in every consumptive person, the blood is highly arterialized and abounds in fibrin. The statement of some of the most distinguished pathologists is, that during the whole process of tubercular disease, when it ends fatally, it depends upon this *fibrous* state of the blood; and, that we cannot cure the disease till we can change this state, and bring back the *venous* condition in which the blood usually exists in a healthy person.

"The idea I mean to communicate is this: alcohol does not nourish the body; and, consequently, would do injury in health; but, it enters, undigested into the circulation, and changes the fibrinous condition of it, (where it exists; but may do great injury in a healthy man, by inducing other diseases, for sometimes people die without consumption,) and thus destroys the *aliment* on which *tuberculization* depends; thus staying consumption, as pregnancy does in the female, and as chronic bronchitis and disease of the heart do, both among men and women. Pregnancy stops, or, if you please, cures consumption, till after the birth of the child, and chronic bronchitis often does the same. If the same state of venous blood continued, after the birth of the child, which existed during pregnancy, consumption would make no progress. It would be forever *stayed*. Now, if we can produce this state of the blood by alcohol, will not consumption be stayed, or cured by its use?

"I would not encourage *intemperance*. I would prescribe alcohol in consumption, as I would *medicine* in any case, and much sooner than I would some other medicines sometimes used in this disease. *But what has a man in health to do with medicine?* The alcoholic lotion used by M. Hall, and which I have used for several years, owed its salutary effects to the *absorption* of the alcohol, in my opinion; and similar good results may be had from its internal use in this disease. I use it both externally and internally in consumption.

"One of the most skillful physicians I ever knew said to me, more than thirty years ago, 'If I had a scrofulous or consumptive patient, his medicine should contain half a pint of rum a day.' But, no man should prescribe this for himself, lest he induce a fatal disease, and die before his time."

So much from my book, published nearly twenty years ago; and, though I am of the same opinion now, as to the use of this drug, in *true tubercular consumption*, yet such is the tendency in the community for stimulants, and among some honest physicians to prescribe them, and such the *mistakes* of others as to the *diagnosis* of phthisis, that I have not a doubt but that much injury has been done by the use of alcohol ostensibly for this disease within the last twenty years.

But what then? Are we to relinquish a remedy, indicated in a certain disease, and which we have found by experience to be useful, because unprofessional men abuse it, or because some physicians ride it as a *hobby*, and prescribe it for all diseases, or because others mistake the disease, and prescribe it when they would not, but for their ignorance in pathology? Suppose we were to subject opium or quinine to this test, would the profession be willing to do it? There are thousands who get intoxicated every day by opium; must our profession, therefore, cease to prescribe opium as a medicine?

I fully concur in the opinion of Dr. Wilks, that "its administration should be watched with the extremest care." I even go further than he does. I have seen some most egregious mistakes of the administration of alcohol, and that even by medical men. It was my fortune, some years since, but after the book above referred to was published, to be on several boards of trust with a member of our profession, well advanced in life, a most worthy man, and one who had refrained wholly for many years from the use of alcoholic liquors, though when young he was in the habit of using them freely. I observed, as I associated with him from week to week, that his health was failing, and I said, "Doctor, you do not look well." He replied, "I am not; my strength has been failing for some months, and I am under medical treatment," and he named his medical adviser, a respectable man in the profession. "He has advised me to take *whisky*, and I have been doing it for some time." I inquired, "what do you take it for? You have no tubercular disease." He said, "no, I have no difficulty of the lungs." In fact, he was as far from being a consumptive as a man could well be. He was short, thick-set, broad chested, and in form, a fair candidate for apoplexy, but not for consumption. I said, "I would leave off whisky, Doctor." He replied, "my friend prescribed it, I suppose, because he thought at my age, being seventy, it would do me good." He continued the whisky, and soon died. A *post-mortem* revealed the fact that his death was caused by *chronic inflammation of the stomach!*

I do not suppose such palpable mistakes as this are often made; yet there are many who prescribe whisky for they know not what, as it was in the case of my old friend and colleague.

I have seen some cases very much like the ones which Dr. W. describes in the following language: "Often have I been called to see a patient apparently dying, etc. [The reader can turn to the description.] In all such cases it is astonishing that any physician should be so ignorant, or so unprincipled. In all nervous diseases, (and they are legion,) in all affections of the liver, diseases of the heart, irritation or inflammation of the stomach, and in health, alcohol has a pernicious effect. Indeed, I know of no case in which I would prescribe it, save in phthisis, and in cases where the fever suddenly leaves a patient so debilitated that he would speedily sink without stimulation."

You have seen, in the long examination of witnesses before a committee of the Massachusetts Legislature, last spring, for and against a license law, what various opinions were expressed upon the use of alcohol as a medicine at the present time. Rev. Dr. John Todd, of Pittsfield, said: "It is a matter of fact that physicians in this city, (Boston) and in my place, prescribe alcohol *ten fold* more than they did twenty-five years ago, and, I think, more than they did forty years."* There never was a grosser mistake than this, which was set right by Prof. Storer, of the Berkshire Medical Institute, who said, "formerly it was the custom for physicians very largely to prescribe alcohol; now a-days, it is not so much the custom. Alcohol was formerly used constantly by physicians; now it is used less. Watery extracts are now used to avoid the use of alcohol."

---

* Alcohol, as a remedy for tuberculosis, was hardly known, even ten years ago; it came suddenly into fashion about that time. Prof. Austin Flint then spoke of it as a new remedy for consumption before the Buffalo Medical Association, and approved its use. Twenty-five years ago alcohol was talked of a medicine, but largely used as a beverage without medical advice, by both the healthy and diseased. Were not Dr. Todd and Dr. Storer both correct? Is not alcohol prescribed "*ten fold*" more now than twenty-five years ago? Are not physicians *now*—that is, for the last two years—beginning to doubt its universal application more than formerly—six or eight years ago?

